# Variables that explain disordered eating behaviors among women: the mediating role of body dissatisfaction

**DOI:** 10.1007/s40519-023-01626-4

**Published:** 2024-01-05

**Authors:** Karina Franco-Paredes, Felipe J. Díaz-Reséndiz, María Angeles Peláez-Fernández, María Leticia Bautista-Díaz

**Affiliations:** 1 Behavior, Health and Quality of Life Academic Group, Centro Universitario del Sur, Universidad de Guadalajara, Av. Enrique Arreola Silva 883, Colonia Centro, Ciudad Guzmán, 49000 Jalisco México; 2 Behavioral Analysis Laboratory, Centro Universitario del Sur, Universidad de Guadalajara, Ciudad Guzmán, Mexico; 3 Department of Social Psychology, Social Work and Social Services, and Social Anthropology, University of Málaga, Málaga, Spain; 4 Bachelor’s Degree Program in Psychology, Facultad de Estudios Superiores Iztacala-UNAM, Tlalnepantla, Mexico

**Keywords:** Body dissatisfaction, Sociocultural influences, Anxiety, Women

## Abstract

**Purpose:**

To analyze the role of body dissatisfaction in the relationships of sociocultural influences, depression, and anxiety with disordered eating behaviors (DEB) in a sample of female Mexican university students.

**Methods:**

A nonrandom sample of 526 female Mexican university students aged 18 to 25 years completed the Questionnaire of Influence on the Aesthetic Model of Body Shape (CIMEC-26), the Hospital Anxiety and Depression Scale (HADS), the Body Shape Questionnaire (BSQ-8D) and the Eating Attitudes Test (EAT-26).

**Results:**

Through the mean model (χ^2^/*df* (5, *n* = 526) = 7.298, *p* = .199; NFI = .996; CFI = .999; RMSEA = .030; SRMR = .011), body dissatisfaction was found to mediate the relationships of influence of advertising, influence of social models and anxiety with DEB (restrictive dieting and bulimia). The variable with the most direct effect on restrictive dieting and bulimia was the influence of advertising. Body dissatisfaction partially mediated this relationship, as the influence of advertising had a significant direct effect on restrictive dieting and bulimia. The final model of direct and indirect effects explained 43% and 22% of the variance in restrictive dieting and bulimia, respectively.

**Conclusion:**

The present study showed that body dissatisfaction partially mediated the relationships between influence of advertising, influence of social models, and anxiety with DEB among women. Thus, these variables should be taken into account in prevention and intervention programs targeting BED. *Level V*: Evidence obtained from a cross-sectional descriptive study.

**Level V:**

Evidence obtained from a cross-sectional descriptive study.

## Introduction

Disordered eating behaviors (DEB) are defined as actions that are performed with the purpose of controlling body weight and shape; for example, restrictive dieting, binge eating, prolonged fasting, skipping meals, self-induced vomiting, and consumption/abuse of laxatives, diuretics or pills not prescribed by health professionals. These behaviors are displayed less often or are less severe than those observed in people with an eating disorder [1, 2]. In Mexico, studies have demonstrated that the prevalence of DEB in women fluctuates between 5.3 and 26% [2–7]. A higher prevalence has been noted in female individuals aged between 15 and 19 years compared with girls aged between 10 and 14 years [5].

Research has been devoted to understanding the factors contributing to DEB. In particular, body dissatisfaction (BD), which is defined as a negative assessment by a person of the size and shape of his or her body, is strongly associated with DEB [8]. Research in Mexico has revealed that between 11 and 69% of young women exhibit BD; specifically, they wanted to be thinner [4, 9, 10]. BD is a robust predictor of behaviors aimed at controlling one’s weight, such as dieting and bulimia [11, 12]. In fact, the presence of BD increases the risk of DEB by 3.6 times among university students [7]. Early evidences showed that sociocultural influences (e.g. influence of advertising, influence of social models, parents and peers) can increase body dissatisfaction which in turn increase the risk for DEB [12]. Additionally, studies have revealed that the internalization of the aesthetic model of thinness increases the risk of developing DEB by 11.8 times in Mexican girls aged 14–18 years [13], and the influence of advertising was related to BD among university women [14].

Cross-sectional and longitudinal studies have shown that, in addition to sociocultural influences, personal characteristics can explain the presence of BD and DEB, such as perfectionism, self-esteem, anxiety, and depression [15]. Adolescents with high scores on depressive symptoms are 1.16 times more likely to develop DEB [16]. Among university students, DEB was associated with the presence of symptoms of depression [7, 17–19] and anxiety [20–23]. Additionally, the interaction between anxiety and students’ place of residence explained the risk of bulimic behavior [24]. Increased depression symptoms have also been linked to higher BD among women [18, 25]. In fact, women experience a significant increase in anxiety and depression after being exposed to articles and images promoting the thinness ideal [26].

To identify variables associated with the presence of DEB, different models have been proposed that offer useful etiological frameworks to examine how sociocultural influences are involved in the development and maintenance of DEB. For example, Thompson et al. [27] developed the tripartite influence model (TIM). This model proposes that sociocultural agents lead to the internalization of the socially constructed ideal body figure and comparison of one’s own body image with the ideal or physical appearance of others. As these comparisons are often unfavorable, the risks of BD and DEB increase. Indeed, 20 years after the TIM was developed, several studies have supported the original proposal of Thompson et al. [27]. In general, the TIM has been corroborated among university students [1, 28–32]. However, some relationships have demonstrated to be nonsignificant in a cross-sectional studies. For example, among female Australian university students (*n* = 464; mean age 20.88 years old), the relationship between BD and restrictive dieting was significant. However, among Malaysian women (*n* = 402; mean age 20.63 years old) this relationship was not significant [31]. Among female French university students (*n* = 190; 18–24 years, mean age 20.70 years), BD was associated with bulimic behaviors. However, this relationship was not found in their Australian (*n* = 188; 18–21 years, mean age 19.56 years) counterparts [30]. These differences can be partially explained by cultural factors, and also by methodological variations of the studies, such as sample size and applied instruments.

The cross-sectional studies also have evidenced directly that BD plays an important role as a mediating factor between DEB outcomes and other independent variables such as identity formation among adolescents and emerging adults (*n* = 327 adolescents, aged 15–18 years, and *n* = 332 emerging adults; aged 18–30 years) [33]; the effect of smartphone addiction among college students (*n* = 5986; aged 15–18 years, mean age 19.8) [34], and body image victimization among adolescents (*n* = 1399; aged 11–17 years, mean age 13.1) [35].

**Fig. 1:**
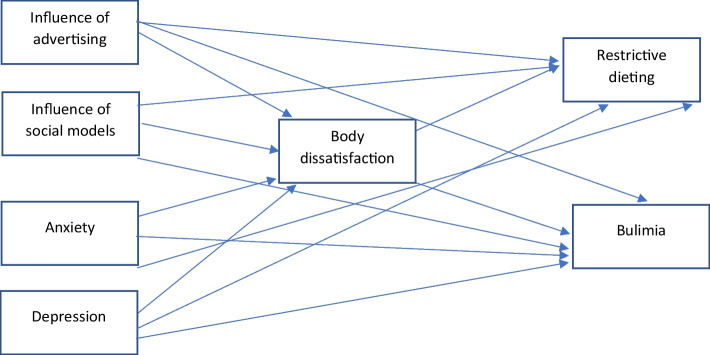
Conceptual model of the mediation analysis

As mentioned above, empirical research has demonstrated that, in addition to sociocultural influences, other variables (e.g., anxiety and depression) are related to DEB. However, the potential mediating role of BD in the relationships of sociocultural influences, anxiety, and depression with DEB has not yet been tested. Likewise, both BD and DEB are culturally specific in their rate, their intensity and predictor factors [30, 36]; therefore, it is necessary to evaluate these variables in each culture. In addition, to the best of our knowledge, this relationship has not yet been explored among female Mexican university students. Understanding the factors that contribute to DEB would inform the development of prevention and intervention programs for this population. Therefore, this study analyzed the role of BD in the relationships of sociocultural influences, depression, and anxiety with DEB in a Mexican sample of female university students. We considered several variables included in the TIM. Accordingly, we hypothesized that BD mediates the relationships of sociocultural influences, depression, and anxiety with DEB, specifically restrictive dieting and bulimia (see Fig. 1).

## Methods

### Participants

A nonrandom purposive sample of 526 women aged 18 to 25 years (*M* = 19.09 years, *SD* = 1.30) enrolled in a public university in Southern Jalisco, Mexico, participated voluntarily in this study. The sample comprised students from nutrition (24.7%), psychology (23.6%), veterinary medicine (24.2) and medicine (27.5) degrees and they were attending the first (63%) and second year (47%) at the university and mostly financially dependent on their families. They belonged to medium–low (28%), medium (32.3%) and medium–high (30.4%) socioeconomic levels.

### Instruments

#### Body Shape Questionnaire (BSQ-8D)

The BSQ-8D is a short version of the BSQ-34 [37] designed to assess dissatisfaction due to weight and body shape. It includes 8 items that are rated on a 6-point Likert scale (from 1 = never to 6 = always). Evans and Dolan [38] proposed this version, which has better psychometric properties than the other short versions. In female samples of Mexican participants, this version has been demonstrated to be one-dimensional, with convergent validity and adequate internal consistency (*α* = 0.89, *Ω* = 0.89) and test–retest reliability (ICC = 0.80) [39, 40]. In the current study, the internal consistency was *α* = 0.92.

#### Eating Attitudes Test (EAT-26)

The EAT-26 [41] evaluates DEB associated with eating disorders (ED) through 26 items on a 6-point Likert scale (never = 0, rarely = 0, sometimes = 0, often = 1, very often = 2, and always = 3). Item 25 is reverse-scored. The Spanish version of EAT-26 has been used [42] to assess a Mexican sample of female university students. This version exhibited adequate internal consistency (*α* = 0.83) and a three-factor structure [43]. In the present study, the internal consistency for the three factors was Restrictive Dieting = 0.80, Bulimia = 0.72, and Oral Control = 0.56. As the internal consistency of the Oral Control factor was low, it was excluded from the analysis.

#### Questionnaire of influence on the aesthetic model of body shape (CIMEC-26)

The 26 items of the CIMEC-26 measure the influence of the aesthetic model of thinness [44] on a 3-point Likert scale (0 = never, 1 = sometimes, and 2 = always). Gómez [45] validated the use of this version in Mexican women and reported an adequate internal consistency for the total score and factors. In the present study, the internal consistency of the two factors were as follows: the Influences of Advertising (α = 0.88), the interest aroused by adverts for sliming products; and the Influence of Social Models (α = 0.81), the interest aroused by the bodies of actresses, fashion models and passers-by in the street.

#### Hospital Anxiety and Depression Scale (HADS)

The HADS [46] evaluates symptoms of anxiety and depression using 14 items scored on a 4-point Likert scale (from 0 = absence of symptoms to 3 = greater presence or intensity of symptoms). Castro-López and Franco-Paredes [47] adapted and validated this scale for use with Mexican youth and established the reliability of anxiety (α = 0.81, Ω = 0.80) and depression (α = 0.76, Ω = 0.72). In a sample of Mexican women with ED, the internal consistency was α = 0.87 [48]. In the present study, the internal consistency was α = 0.86 for anxiety and α = 0.74 for depression.

### Procedure

Permission was requested from the teachers of each group to invite students to participate in the research. One of the members of the research team connected to the online classes to explain the purpose of the research and to invite the students to participate voluntarily in the research. After learning the purpose of the study, informed consent was obtained from all participants. The participants completed the questionnaires individually at the beginning of their online classes via the platform Meet. A researcher remained online to answer any questions that arose and prevent bias in responses among participants. Students received no reimbursement or gifts for their participation. No reports were received of difficulties in understanding the items, and participants commented that they felt good while answering the questionnaires. The research was approved by the Ethics Committee of the CUSur at the University of Guadalajara and was carried out in strict compliance with the Psychologist’s Code of Ethics [49] and the principles of the Declaration of Helsinki. This was a low-risk study according to Mexican norms [50].

### Analytical strategy

A descriptive analysis of the data was first carried out using SPSS ver. 26 (IBM, Armonk, NY, USA). Second, Pearson’s correlation analyses were used to explore the relationships of anxiety, depression, social influence, and advertising influence with BD, restrictive dieting, and bulimia. Finally, a structural equation model in SPSS AMOS 25 was constructed to analyze the mediation effect of BD on the relationships of anxiety, depression, social influence, and advertising influence with restrictive dieting and bulimia.

## Results

Table 1 presents the descriptive statistics and Pearson correlation coefficients of the scales used. As expected, anxiety, depression, social influence, and advertising influence correlated significantly and positively with BD, restrictive dieting, and bulimia.

**Table 1:** Mean, standard deviation, internal consistency and intercorrelations between study variables

	Min	Max	M	SD	1	2	3	4	5	6
1. Restrictive dieting	0	27	4.27	5.29						
2. Bulimia	0	12	0.87	1.76	0.584^***^					
3. Body dissatisfaction	8	48	22.37	10.01	0.620^***^	0.433^***^				
4. Anxiety	0	21	9.25	4.53	0.345^***^	0.335^***^	0.466^***^			
5. Depression	0	16	4.90	3.33	0.241^***^	0.276^***^	0.389^***^	0.639^***^		
6. Influence of advertising	0	24	4.50	4.59	0.568^***^	0.369^***^	0.643^***^	0.408^***^	0.329^***^	
7. Influence of social models	0	12	4.18	2.97	0.494^***^	0.377^***^	0.698^***^	0.390^***^	0.342^***^	0.628^***^

***p < 0.01

**Fig. 2:**
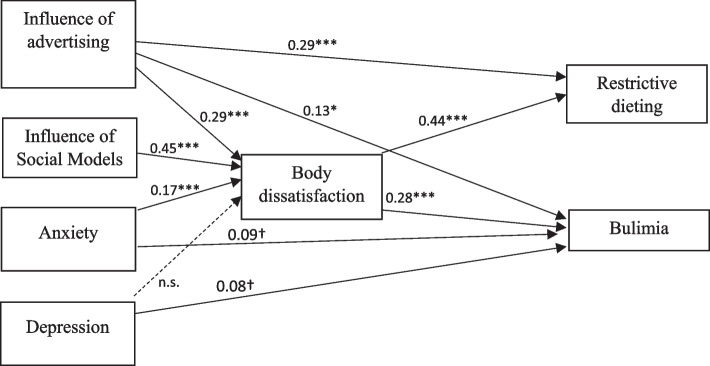
Final mediation model: standardized estimates of direct effects of influence of advertising, social influence, anxiety and depression on restrictive diet and bulimia through body dissatisfaction. *n.s.* non-significant; †*p* < 0.10; *p < 0.05; **p < 0.01; ***p < 0.001

The structural equation model included BD as a mediator of the relationships of anxiety, depression, social influence, and advertising influence with restrictive dieting and bulimia. Depression did not significantly influence BD. However, the associations of anxiety and depression with bulimia approached significance (*p* < 0.10). The final model (see Fig. 2) revealed that BD was a significant mediator of the relationships of anxiety, social influence, and advertising influence with restrictive dieting and bulimia; it also demonstrated satisfactory fit with the data: χ^2^/*df* (5, *n* = 526) = 7,298, *p* = 0.199; normed fit index [NFI] = 0.996; comparative fit index [CFI] = 0.999; root mean squared error of approximation [RMSEA] = 0.030; and standardized root mean squared residual [SRMR] = 0.011. The final mediation model of direct and indirect effects explained 43% and 22% of the variance in restrictive dieting and bulimia, respectively.

### Path coefficients of the model

Table 2 summarizes the results of the multiple mediation analysis, indicating the path coefficients and confidence intervals for each effect tested in the model. The associations of anxiety, social influence, and advertising influence with restrictive dieting and bulimia were mediated by BD (b). BD only partially mediated the relationships of advertising influence with restrictive dieting and bulimia, as the direct effect (a) was statistically significant in this analysis.

**Table 2:** Path coefficients and confidence intervals from mediation analyses

Independent variables (IV)	Mediating variables (M)	Dependent variables (DV)	Direct effect of IV on M	Direct effect of M on DV	Direct effect of IV on DV	Indirect effect of IV on DV	95% CI for indirect effect	Total effect
Influence of advertising	Body dissatisfaction	Restrictive dieting	0.29***	0.44***	0.29***	0.13***	0.088–0.167	0.41***
Influence of social models			0.45***		0.00	0.20***	0.138–0.261	0.20***
Anxiety			0.17***		0.00	0.08***	0.043–0.113	0.08***
Depression			0.00		0.00	0.00	0.00–0.00	0.00
Influence of advertising	Body dissatisfaction	Bulimia	0.29***	0.28***	0.13*	0.08***	0.043–0.123	0.21**
Influence of social models			0.45***		0.00	0.13***	0.064–0.190	0.13***
Anxiety			0.17***		0.09	0.05***	0.022–0.080	0.14*
Depression			0.00		0.08	0.00	0.00–0.00	0.08

Estimated using bias corrected and accelerated bootstrapping, with 5000 samples; *CI* confidence interval

*p < 0.05; **p < 0.01; ***p < 0.001

## Discussion

This study analyzed the role of BD in the relationships of sociocultural influences, depression, and anxiety with DEB in a Mexican sample of female university students. The model displayed adequate fit the findings showed that sociocultural factors (influence of advertising and influence of social models) and anxiety explained two DEBs (restrictive dieting and bulimia) through body dissatisfaction. Consistent with previous research, advertising and influence of social models, which promote the ideal of thinness, were associated with BD and DEB in female university students [51, 52]. Notably, influence of social models (i.e., the influence of artists and models appearing in the media and passers-by in the street) had the greatest effect on BD; in turn, BD had the greatest effect on restrictive dieting. This influence has been widely documented in previous studies with university students [28–30, 53]. In Mexico, there is evidence of relationships of sociocultural influences with BD and DEB in women, highlighting that the influence of the body aesthetic model increases the risk of DEB [13]. These results confirm that the global influence of the aesthetic ideal, enhanced by digital media, equates female success with thinness and leads women to be dissatisfied with the shape and size of their body. Sociocultural influences have contributed to the normalization of thinness; therefore, the risks it poses to both physical and mental health are overlooked, highlighting the importance of such studies [54].

Particularly, BD only partially explained the relationships of advertising influence with DEB, restrictive dieting, and bulimia, as the direct effect was statistically significant. This implies that pro-thinness advertising alone explains the presence of DEB. This finding is in line with that of previous research [52]. The influence of the thinness model is increasingly well documented. A number of correlational, experimental, and prospective studies support that the thinness ideal is related to BD and DEB [7, 52]. Therefore, it is necessary to exert widespread and collective efforts to counteract the influence of advertising and the influence of social models to which women are exposed. The introduction of the internet and digital media have increased the negative effects of the thinness ideal on people [55].

An interesting finding was the effect of anxiety in the model. A decade earlier, a study documented that Spanish patients with EDs had higher anxiety scores than the control group and that anxiety increased the risk of developing these disorders tenfold or more [56]. In the Mexican population, a significant effect of the interaction between sex, zone of residence (bordering the United States or in the center of the country) and anxiety on bulimia nervosa was observed [24]. Although there is evidence of a relationship between anxiety and ED, in this study, the influence of anxiety and that of the aesthetic model of thinness (advertising and influence of social models) were evaluated simultaneously; all were related to BD and DEB. Finally, direct effects of depression on BD and DEB were not observed. A possible explanation is that scores of the university student’s women who participated in this study reflect that their symptoms of depression are mild, unlike anxiety, because the last one indicating must be attended by professional psychologist.

### Strengths and limitations

This study has theoretical and practical implications. At a theoretical level, these findings shed light on the effect of BD on the relationship between social influence and influence of advertising and DEB among Mexican women. Specifically, influence of advertising contributed both individually and jointly to restrictive dieting and bulimia. Therefore, theoretical approaches regarding the effects of influence of advertising on DEB among this population could incorporate BD as a potentially relevant variable. Regarding practical implications, prevention and intervention programs aimed at reducing DEB in this population might benefit from including strategies focused on increasing body satisfaction and criticizing the beauty models promoted by the media.

This study has several limitations. First, it has a cross-sectional design; therefore, it was not possible to make inferences regarding causal relations. Future research should employ longitudinal and experimental designs. Second, the sample consisted of university students, which limits the generalization of the findings to clinical samples. Further studies including samples with diagnosed EDs could allow generalization of the results to clinical populations. Third, this study used self-reported data; thus, the findings may be subject to desirability and variance biases. Future research using complementary clinical interviews or performance measures would reduce this type of bias.

These findings suggest three main directions for future studies. First, it is necessary to assess the unique pressures experienced by women in specific age groups (adolescents, youth, and adults) as well as men. Second, studies should determine intervention and prevention strategies for university students to reduce advertising and social models influence and to promote protective factors such as body acceptance and a positive body image. Third, further research on this model is needed in the Mexican population to confirm or refute our findings.

## Conclusions

The present study found that BD mediates the relationship of sociocultural influences (advertising and of social models influence) and anxiety with DEB in female Mexican university students. Advertising influence had a direct effect on restrictive dieting and bulimia. Therefore, it is recommended that intervention programs consider the influence of advertising. In addition, this study highlights the importance of analyzing the model in specific populations to identify additional relationships not included in the original model.

### What is already known on this subject?

Previous research has identified a relationship between body dissatisfaction and disordered eating behaviors among university students. Specifically, higher levels of body dissatisfaction are related to disordered eating behaviors. Additional research is needed to determine whether body dissatisfaction mediates the relationships of sociocultural influences, anxiety and depression with disordered eating behaviors.

### What does this study add?

This study contributes to the literature by identifying some variables that explain the presence of disordered eating behaviors in young women and expand research in female Mexican university students. Specifically, the results showed that body dissatisfaction mediates the relationships of sociocultural influences (advertising and social models influence) and anxiety with disordered eating behaviors in female university students. A mediation model was used to analyze the relationships among these variables. The findings have implications for future research as well as targeted prevention programs in adolescent girls with disordered eating behaviors.

## Data Availability

The datasets analyzed during the current study are available from the corresponding author upon reasonable request.
